# Influence of the initial foot contact strategy on knee joint moments during stair and ramp descent

**DOI:** 10.1038/s41598-020-70933-y

**Published:** 2020-08-18

**Authors:** Hyeong-Min Jeon, Eui-Bum Choi, Jae-Hoon Heo, Gwang-Moon Eom

**Affiliations:** Biomedical Engineering, Konkuk University, Chungju-city, 27478 Republic of Korea

**Keywords:** Disease prevention, Public health

## Abstract

Gait modification strategies are effective in reducing knee joint loads, which are associated with the development and progression of knee osteoarthritis (OA). However, the effect of modification of the initial foot contact method in high-loading descending task was not investigated. Here, we show that the initial foot contact strategy significantly alters knee joint moments during descending tasks. We found that the second peak flexion moment was lower for the forefoot strike (FFS) than for the rearfoot strike (RFS) in both stair and ramp descent. As for the peak adduction moment, the second peak was lower for the FFS in stair descent, but two peaks were inconsistent in ramp descent. Our results demonstrate that the knee joint loads can be reduced by simple modification of the initial foot contact strategy. In both descending modalities, the FFS may benefit people with early OA in the patellofemoral joint, whose progression is associated with the peak flexion moment. Likewise, the FFS during stair descent may benefit people with early OA in the medial knee, whose progression is associated with the peak adduction moment. The results would be helpful for prevention and rehabilitation programmes of knee OA.

## Introduction

Osteoarthritis (OA) is the most common joint disease accompanied by chronic pain and disability in developed countries^1,2^, and knee OA accounts for more than 80% of all OA diseases^2^. Knee OA induces pain and stiffness in the knee, thus decreasing its range of motion^3,4^; furthermore, knee OA progression leads to disorders that disrupt daily activities^5^. Approximately 10% of older adults (> 55 years) have painful disabling knee OA, of whom one-quarter are severely disabled^6^.

Increased mechanical load plays an important role in the initiation and progression of knee OA, contributing to the deterioration and loss of the articular cartilage matrix^7,8^. In particular, mechanical loading during repetitive weight-bearing activities such as walking was shown to be important for the progression of knee OA^9–14^, where the external joint moment was used as a biomechanical indicator of such loading. Specifically, an increase in the peak and impulse of the external knee adduction moment was associated with OA at the tibiofemoral joint of the medial knee^9–11^, i.e. they were associated with the severity of OA and varus malalignment^11^ and with the loss of the medial cartilage 1–5 years later^9,10^. Meanwhile, an increase in the peak and impulse of the external knee flexion moment was associated with OA at the patellofemoral joint of the knee^12–14^, i.e. they were associated with the existence of OA^13^, worsening of cartilage health^14^, and progression of OA after 1 year^12^.

Thus, reduction in the external knee flexion and adduction moments during walking would be beneficial for individuals with early OA because it may slow down knee OA^15^. Descent walking on stairs and ramps, which is frequently encountered in daily activities, results in an external knee flexion moment that is 2–7 times that generated during level walking^16,17^. Moreover, the descending task significantly increases the knee load compared to an ascending one, i.e. the first peak adduction moment is 1.6 times that obtained during stair ascent^18^. Therefore, a strategy to reduce the knee moments during descent walking would be important.

Gait modification strategies have been attempted to reduce knee joint moment. Such strategies were based on the mechanical perspective that alteration of one joint (e.g. by foot posture) can influence the adjacent joints through linkage dynamics. The strategies include deliberate modification of the foot progression angle (on the transverse plane) into excessive toe-in or toe-out posture^19–21^, increasing the step width on the frontal plane^22,23^, and using a combination of the toe-in strategy and wider step width^21^. For example, the peak adduction moment decreased, but the peak flexion and rotation moment increased during level walking in the toe-in and toe-in with wider step strategies^21^. Furthermore, the second peak decreased but the first peak increased during stair ascent in the toe-out strategy^15^.

It can be also anticipated from the linkage dynamics that the modification of the initial foot-contact patterns in the sagittal plane also influences the knee joint moments during descending gait. In fact, the foot contact strategy significantly affected the knee joint load during running^24–26^. The foot-strike patterns were categorised depending on the portion of the foot that initially contacts the ground, which include the forefoot and rearfoot strike (FFS and RFS, respectively)^24^. In case of running, FFS increased the knee joint moments and contact forces than the RFS^25^.

However, there has been controversy about the better foot-strike strategy of descending gait. Some physical therapists recommended that the FFS (with more power absorption at the ankle joint) would reduce the knee joint load during stair descent, whereas some emphasised strengthening of knee muscles in the supporting leg without any specification of the foot-strike method in the leading leg. Nevertheless, there was no systemic biomechanical evidence based on experiments in their claims. Furthermore, the effect of foot-strike patterns on the knee joint load may differ in different descending environments such as stairs and ramp. Therefore, this study aimed to investigate the effect of the initial foot contact strategy on the knee flexion and adduction moments during stair and ramp descent.

We defined two foot-strike strategies of descending gait. In the RFS gait, the initial contact on the ground is made with the central aspect of the heel (no varus or valgus) and eventually toeing off as in the normal walking with a heel strike. In contrast, in the FFS gait, the initial contact is on the forefoot (the area spanning from the metatarsal heads to the toes), but the heel does not touch the ground during initial contact.

## Results

**Figure 1:**
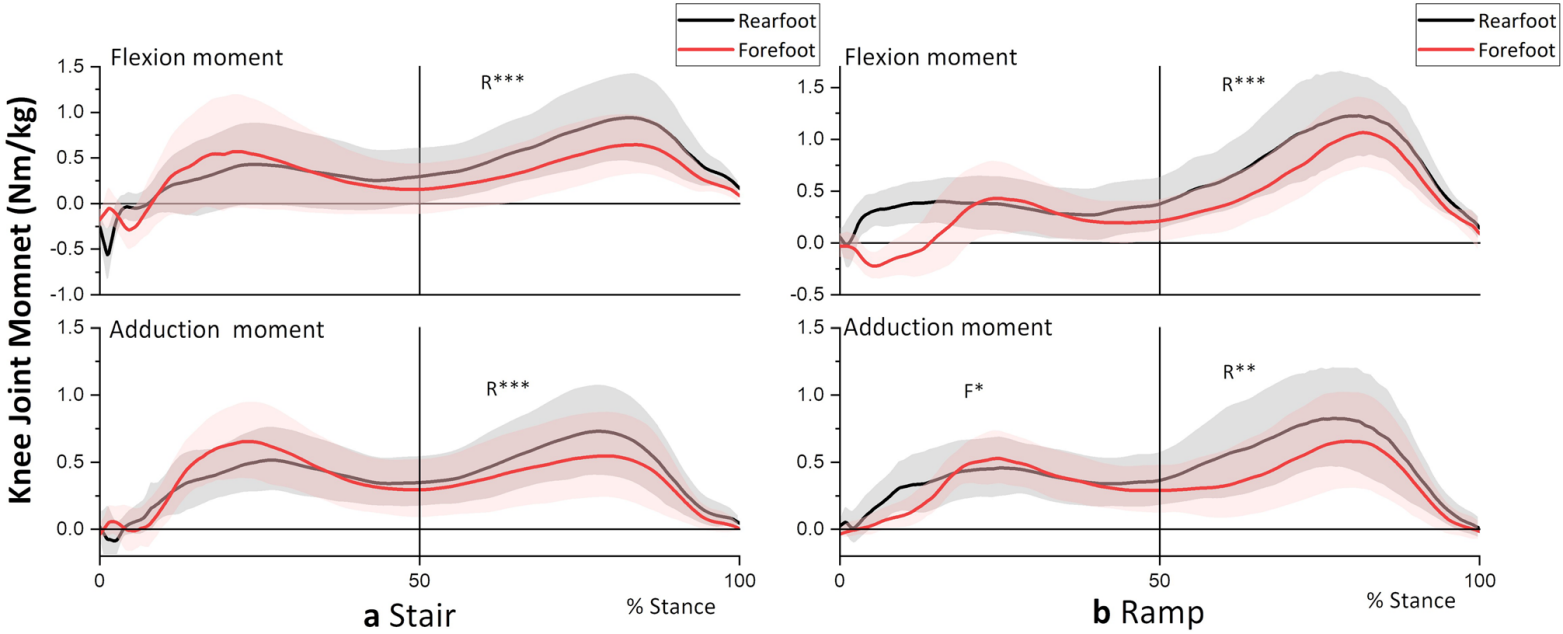
Knee joint external moment during (**a**) stair and (**b**) ramp descent. R and F indicate a significantly greater peak moment for the RFS and FFS, respectively. *P < 0.05, **P < 0.01, ***P < 0.001.

**Figure 2:**
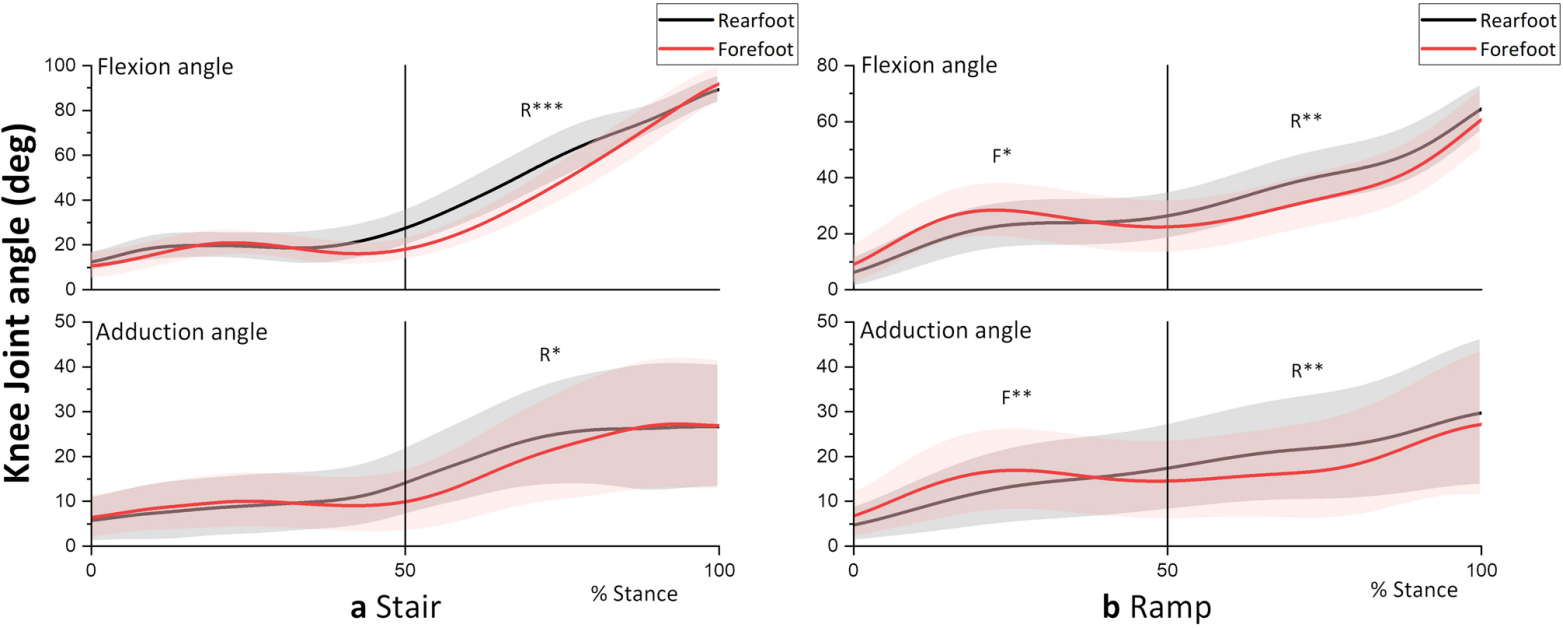
Knee flexion and adduction angle during (**a**) stair and (**b**) ramp descent. R and F indicates a significantly greater joint angle for the RFS and FFS, respectively, at the corresponding moment peak. *P < 0.05, **P < 0.01, ***P < 0.001.

**Figure 3:**
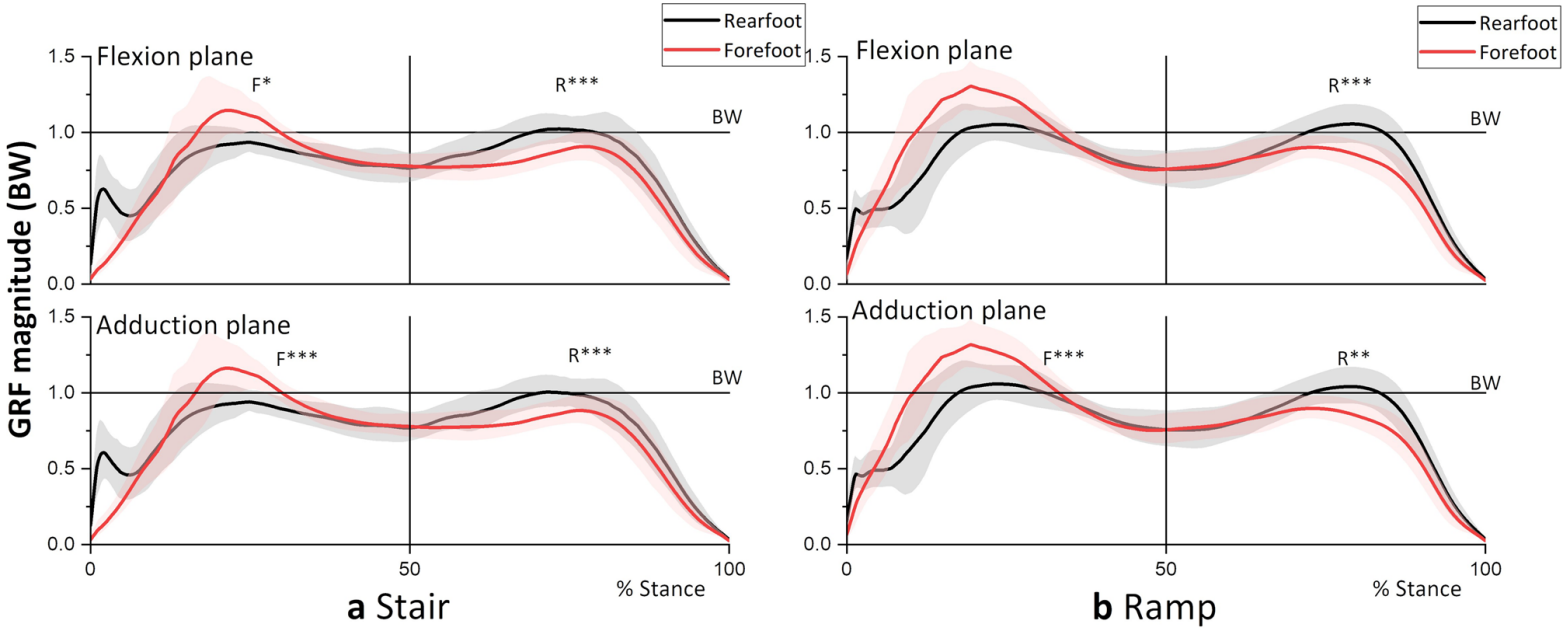
Magnitude of the GRF during (**a**) stair and (**b**) ramp descent. R and F indicate a significantly greater GRF for the RFS and FFS, respectively, at the moment peaks of the corresponding plane. *P < 0.05, **P < 0.01, ***P < 0.001.

**Figure 4:**
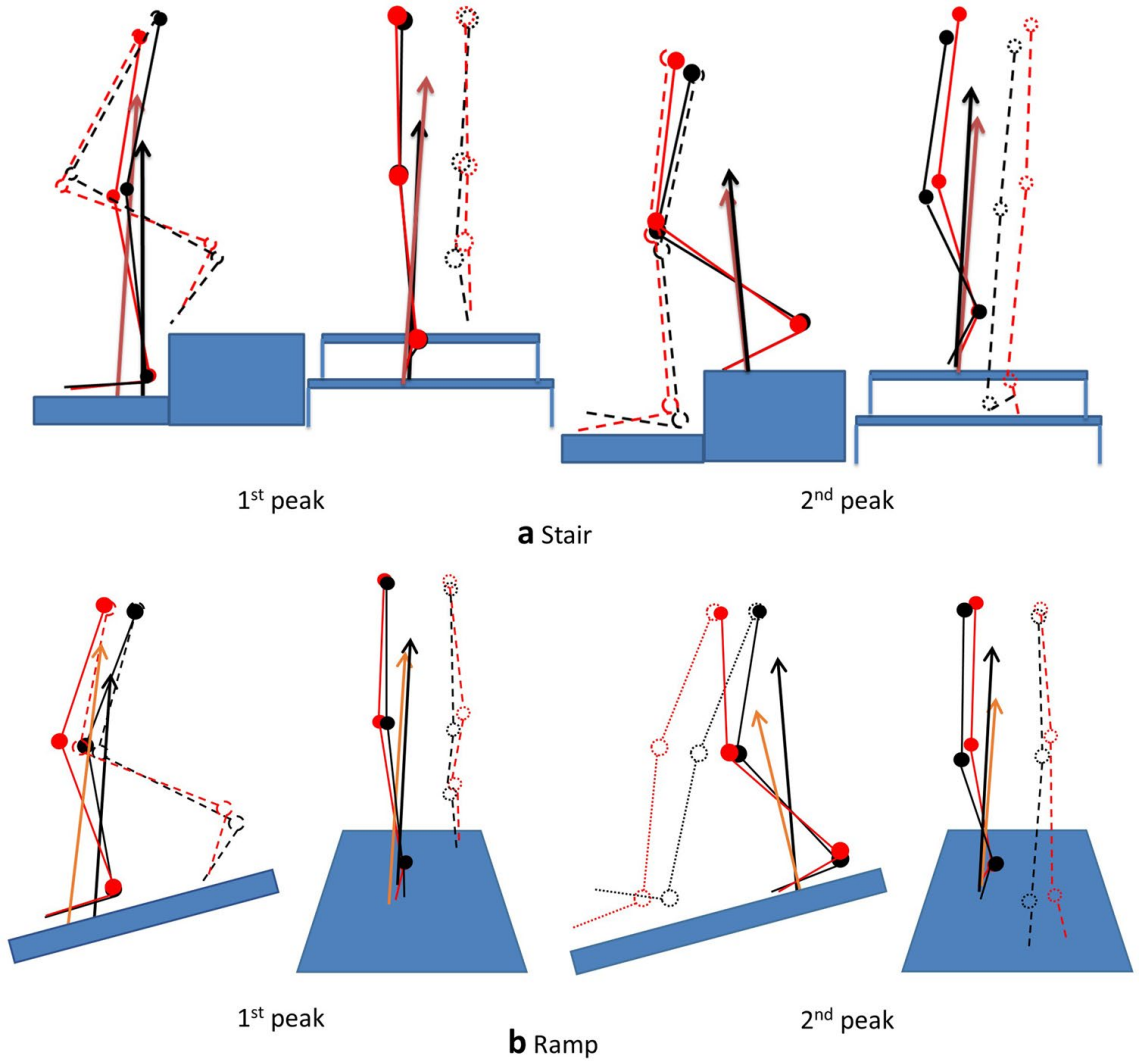
Stick figures in the sagittal and frontal planes at instances of peak knee flexion and adduction moments (extracted from the data of a representative participant): (**a**) stairs and (**b**) ramp. The black and red colours represent the rearfoot and forefoot strike strategies, respectively. The solid and dashed lines represent the ipsilateral and contralateral legs, respectively. The arrows represent the GRF vectors.

Figures 1, 2, and 3 show the mean [with standard deviation (s.d.)] knee joint external moments, knee joint angles, and magnitude of ground reaction force (GRF), respectively, for 19 participants. Table 1 shows local feature variables in the first and second halves of the stance (knee joint moment, moment arm, and GRF magnitude) compared between foot contact strategies. Figure 4 shows stick figures at instances of knee joint moment peaks. Table 2 compares global feature variables (global peak moments and angular impulses) between the FFS and RFS gait. Table 3 compares spatiotemporal features between the FFS and RFS gait.

**Table 1:** Comparison of local feature variables (determined separately from the first and second halves of stance) in different foot contact strategies.

Task	Time instance	Direction	Feature (unit)	Rearfoot strike		Forefoot strike	P value
Stair descent	First moment peak	Flexion	Peak moment (Nm/kg)	0.54 ± 0.19		0.52 ± 0.31	0.65
			Moment arm (mm)	62.1 ± 29.8	>	45.0 ± 21.7	**
			GRF magnitude (BW)	0.82 ± 0.25	<	1.04 ± 0.28	*
		Adduction	Peak moment (Nm/kg)	0.53 ± 0.21		0.59 ± 0.18	0.12
			Moment arm (mm)	61.7 ± 19.2		53.2 ± 30.9	0.14
			GRF magnitude (BW)	0.96 ± 0.12	<	1.14 ± 0.16	***
	Second moment peak	Flexion	Peak moment (Nm/kg)	1.32 ± 0.44	>	1.11 ± 0.33	***
			Moment arm (mm)	125 ± 36.4		126 ± 37.0	0.78
			GRF magnitude (BW)	1.01 ± 0.10	>	0.86 ± 0.11	***
		Adduction	Peak moment (Nm/kg)	0.91 ± 0.39	>	0.71 ± 0.37	***
			Moment arm (mm)	93.0 ± 32.7		77.4 ± 65.9	0.60
			GRF magnitude (BW)	1.04 ± 0.07	>	0.88 ± 0.09	***
Ramp descent	First moment peak	Flexion	Peak moment (Nm/kg)	0.52 ± 0.42		0.62 ± 0.6	0.17
			Moment arm (mm)	45.9 ± 35.4		54.1 ± 33.9	0.13
			GRF magnitude (BW)	1.02 ± 0.22		1.09 ± 0.38	0.17
		Adduction	Peak moment (Nm/kg)	0.58 ± 0.22	<	0.70 ± 0.25	*
			Moment arm (mm)	52.1 ± 22.3		57.8 ± 22.1	0.17
			GRF magnitude (BW)	1.05 ± 0.21	<	1.28 ± 0.16	***
	Second moment peak	Flexion	Peak moment (Nm/kg)	0.98 ± 0.49	>	0.68 ± 0.32	***
			Moment arm (mm)	101 ± 41.0		98.2 ± 48.4	0.18
			GRF magnitude (BW)	1.01 ± 0.17	>	0.78 ± 0.20	***
		Adduction	Peak moment (Nm/kg)	0.76 ± 0.34	>	0.60 ± 0.33	**
			Moment arm (mm)	78.3 ± 30.2		74.7 ± 33.4	0.26
			GRF magnitude (BW)	1.05 ± 0.13	>	0.89 ± 0.11	**

The moment arm and GRF magnitude were extracted at instances of knee joint moment peaks (during flexion and adduction). Values are presented as mean ± SD. *P < 0.05, **P < 0.01, ***P < 0.001.

**Table 2:** Comparison of global feature variables (determined from the whole stance period) in different foot contact strategies.

Task	Direction	Feature (unit)	Rearfoot strike		Forefoot strike	P value	RFS > FFS ratio (%)^a^
Stair descent	Flexion	Global peak moment (Nm/kg)	1.32 ± 0.44	>	1.12 ± 0.32	***	84
		Angular impulse (Nms/kg)	281 ± 116	>	203 ± 85.8	***	100
	Adduction	Global peak moment (Nm/kg)	0.91 ± 0.38	>	0.76 ± 0.31	***	79
		Angular impulse (Nms/kg)	222 ± 99.8	>	174 ± 77.9	***	95
Ramp descent	Flexion	Global peak moment (Nm/kg)	0.99 ± 0.49	>	0.81 ± 0.50	*	74
		Angular impulse (Nms/kg)	224 ± 142	>	181 ± 138	**	74
	Adduction	Global peak moment (Nm/kg)	0.80 ± 0.31		0.76 ± 0.28	0.42	
		Angular impulse (Nms/kg)	204 ± 79.2		189 ± 93.6	0.15	

Values are presented as mean ± SD.

^a^Proportion of subjects who showed greater value of the corresponding feature variable for the RFS than for the FFS.

*P < 0.05, **P < 0.01, ***P < 0.001.

**Table 3:** Comparison of spatiotemporal features in different foot contact strategies.

Task	Feature (unit)	Rearfoot strike		Forefoot strike	P value
Stair descent	Step with (m)	0.13 ± 0.04	ns	0.12 ± 0.03	0.49
	Stride length (m)	0.64 ± 0.04	ns	0.65 ± 0.03	0.71
	Walking speed (m/s)	0.63 ± 0.13	<	0.77 ± 0.07	*
Ramp descent	Step with (m)	0.12 ± 0.03	ns	0.11 ± 0.03	0.35
	Stride length (m)	1.43 ± 0.03	ns	14.4 ± 0.09	0.06
	Walking speed (m/s)	0.92 ± 0.07	ns	0.89 ± 0.12	0.57

The walking speed was calculated as the diagonal travel distance per second. Values are presented as mean ± SD. *ns* not significant.

*P < 0.05.

### Stair descent

From Figs. 1a and 3a and Table 1, it is evident that the second peaks of the flexion and adduction moments as well as the GRF magnitude were greater for the RFS than for the FFS (P < 0.001). The result indicates greater loading (GRF) on the supporting trailing limb (with more bended knee, Fig. 2a) in the RFS contributed to the increased peak knee flexion and adduction moments compared to those in the FFS.

In contrast, the first peak moments were not different between strategies (Fig. 1a and Table 1). At the instance of the first peak moments, GRFs on both flexion and adduction plane were greater for the FFS (Fig. 3a, P < 0.001), whereas the moment arm was greater for the RFS (P < 0.01 for flexion and P = 0.15 for adduction), which resulted in no difference in the joint moments.

All global load features were greater for the RFS than for the FFS (P < 0.001), with most participants (79–100%) showing greater values for the RFS (Table 2). The only spatiotemporal difference was the walking speed, where the speed for the FFS descent was 1.2 times that for the RFS (P < 0.05, Table 3).

### Ramp descent

As in the case of stair descent, the major difference in the knee joint moment was in the second half of the stance, where the RFS had greater peaks in the flexion and adduction moments and also GRF magnitude at the peak instance (Fig. 1b and Table 1). Again, the result indicated that greater loading (GRF) on the trailing leg in the RFS contributed to increased knee joint moments.

In the first half of the stance, the peak adduction moment and GRF magnitude of the FFS were greater than those of the RFS (Table 1), which indicates that the greater loading on the leading leg (with more knee bending, as shown in Figs. 2b and 4b) contributed to greater peak knee adduction moments in the FFS.

Global load features in Table 2 show that only the flexion loads were different, where those of the RFS were greater than those of the FFS in 74% of the participants. In contrast to the case of stairs, no spatiotemporal difference was observed between strategies in ramp-descent gait (Table 3).

## Discussion

This study investigated the effect of initial foot contact strategy on the knee flexion and adduction moments when descending stairs and ramp. In the second half of the stance during stair descent, the contralateral leading leg of the RFS strategy needs to be in a lower position than the FFS so that the heel can make contact with the ground (Fig. 4a). This would require more flexion and adduction of the knee in the ipsilateral supporting (trailing) leg until the initial contact of the contralateral leg on the ground, which was shown in the knee angles at instance of peak moments (Fig. 2a). A dorsiflexed ankle at the initial contact in the RFS (Fig. 4a) would reduce shock absorption at the ankle joint compared with that in the FFS (i.e. plantarflexors would absorb greater amounts of power than dorsiflexors). These may have increased reliance on the trailing leg (as shown by the greater GRF in the RFS in Table 1), which contributed to the increase in the flexion and adduction moments of the RFS.

The peak GRF magnitude for the RFS exceeded the body weight (BW) in the second half of the stance on the stairs (Fig. 3a), indicating that deceleration of the BW lowering occurred, and accordingly, a greater knee moment was required. In contrast, the GRF of the FFS was lower than the BW in the second half of the stance, indicating that the BW was accelerated downward or abruptly transferred to the contralateral leg throughout the second half of the stance. This difference in deceleration in the second half of the stance may have resulted in differences in GRF in the first half of the stance, that is, a greater loading (GRF) on the supporting trailing leg (second half) of the RFS would be associated with less loading on the leading leg (first half). Similarly, less loading on the trailing leg (second half) of the FFS would be associated with greater loading on the leading leg (first half). However, the first peak moments of the FFS were not different from those of the RFS, which may be due to the cancellation of the effects by the increased moment arm in the RFS (Table 1).

The fact that all global load features showed greater values for the RFS than for the FFS (Table 2) suggests that walking downstairs with the FFS benefits people with early stages of OA. Specifically, the lower flexion and adduction loads in the FFS may slow down the progression of OA in the patellofemoral^12–14^ and medial tibiofemoral joints^9–11^, respectively. It is noted that 0–21% of the participants showed opposite results (Table 2); thus, caution is needed in the interpretation of the results. A faster speed results in greater peak knee joint moments during stair descent^27^ and level walking^28^. Therefore, the smaller knee loads in the FFS cannot be attributed to its faster walking speed.

Similar to the case of stair descent, the leading leg of the RFS in ramp descent needs to be in a lower position and the hip joint should be more posterior than the FFS (Fig. 4b), which require more knee bending (Fig. 2b) and loading (Fig. 3b) on the supporting (trailing) leg. The GRF of the RFS exceeded the BW (Fig. 3b and Table 1), which indicates that deceleration of BW lowering is performed by the supporting trailing leg. Therefore, the increase in GRF could have contributed to the increase in the second peak moments of the RFS.

Similar to the case of the stairs, GRF loading on the leading leg in the first half of the stance was greater for the FFS (Fig. 3b and Table 1). This abrupt transfer of the GRF to the leading leg (from the trailing leg) in the FFS can be anticipated by a GRF smaller than the BW in the second half of the stance (Fig. 3b) and a more anterior positioning of the center of mass (coming from the anterior positioning of the hip joint) at the instance of the first peak moment (Fig. 4b). The greater loading on the leading leg and tendency for greater moment arm in the FFS could have contributed to a greater first peak adduction moment, but the effect was not significant for the flexion moment.

Greater global knee flexion loads in the RFS (Table 2) suggest that walking down the ramp with the FFS benefits people with early-stage patellofemoral OA^12–14^. It is noted that 26% of participants showed the opposite results (Table 2); thus, caution is needed in the interpretation of the results.

The results of this study do not agree with those of level running^25^, where the external knee flexion moment tended to be greater for FFS runners than for RFS runners in the majority of the stance period. The difference can be attributed to differences between running and walking and between level and descending locomotion. Knee joint moments have only one peak per stride in running but has two peaks in walking. Two peaks in walking may represent weight acceptance and push-off (controlled lowering in case of descent walking), whereas these are overlapped in running. Meanwhile, the first peak is usually greater in case of level walking, in contrast to the greater second peak for descent walking. Therefore, the peak knee moments in level running may have been dominated by weight acceptance, where the FFS has a greater impact force from the ground and, accordingly, greater knee flexion moment. Even in the descent walking in this study, the first peak tended to be greater for the FFS. However, the global peak moments were dominated by the second peak moments in the descent walking this study (Table 2).

In summary, the FFS resulted in lower global peak flexion and adduction moments and impulses in stair descent and lower global peak flexion moment and impulses in ramp descent, which suggests that the FFS could be a rehabilitation strategy for patients with early OA or an OA prevention strategy for normal participants. Supposing that most patients with OA are already adopting the FFS strategy during stair descent, the biomechanical rationale of their choices would have practical value, e.g. as evidence of the recommendation of such strategy in normal individuals and patients with early OA. This would be more applicable for ramp descent, for which the majority of normal individuals adopt the RFS strategy.

In a further study, the present results need to be verified in patients with OA. It would be valuable to investigate whether the effect of the initial foot strike would be similar in the other descending environments, e.g. a more inclined ramp as encountered in downhill hiking. A combination of strategies, such as the FFS with wider step width, is another interesting topic.

## Methods

### Experiments

**Figure 5:**
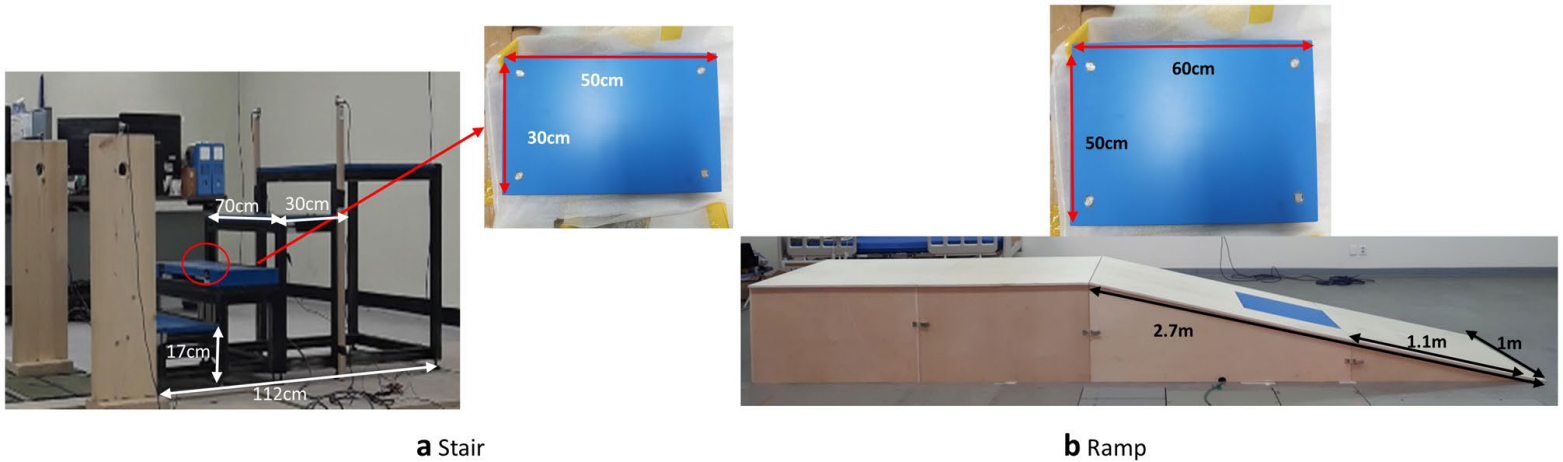
Instrumented (**a**) stairs and (**b**) ramp.

Nineteen healthy young men (age 23.4 ± 1.3 years; height 1.74 ± 0.06 m; weight 72.2 ± 8.6 kg; leg length 90.5 ± 4.7 cm) participated in this study. All participants provided informed consent, and this study was approved by the institutional review board at the Konkuk University, Korea. A motion capture system with 12 cameras (Vicon, UK) with the plug-in-gait marker set and instrumented stairs and ramp (Fig. 5) was used to measure the kinematic and kinetic data. The stairs comprised three standard indoor steps (tread 30 cm; height 17 cm; width 70 cm; inclination 30°), a top platform (70 × 62 cm), and a force plate (9260AA3; Kistler, Switzerland) inserted on the second step. The ramp (length 2.7 m; width 1 m; inclination 15°; top platform 1 × 2 m) was instrumented with one force plate (9260AA6; Kistler) inserted at the centre of the ramp. All force plates were covered with slip-resistant sheets (3M, St. Paul, MN, USA) with the colour same as the stairs and ramp.

The selected initial foot contact strategies were the RFS and FFS. Participants were asked to descend sticking to the definition of each strategy (as described in the “Introduction” section) and to walk naturally at their own comfortable speeds with natural arm swing and to maintain an upright upper body posture (i.e. they should not watch their feet). Consecutive practice trials (> 5 times) were performed for each strategy in each gait environment, because some strategy and environment combinations were unfamiliar to the participants, FFS and RFS walking were measured after enough practice trials of the FFS and RFS, respectively. A foot-strike strategy sequence consisted of practice and measurement trials. The order of foot-strike strategy sequences was randomised, so was the order of gait environments (stairs or ramp). A 10-min rest was provided between the trials to reduce the effect of fatigue. Two successful trials were recorded for each condition, and the trial with more natural walking was used for the analysis. Exclusion criteria of successful trials were varus or valgus foot at initial contact, unnatural or jerky walking determined either by the inspector or the participant, and wrong contact trials determined either by the inspector close to the walking participant or the inspector watching the foot markers and GRF position on a computer screen. All experimental methods were performed in accordance with the relevant guidelines and regulations. This regulations by was approved by the institutional review board at the Konkuk University, Korea.

### Analysis

External knee flexion and adduction moments were calculated through the inverse dynamics based on the dynamic plug-in-gait model provided by the Nexus programme (Vicon). The knee joint angles (flexion and adduction) were also calculated by the Nexus programme. The knee joint angles, called the Tait–Bryan (Cardan) angles^29^, are relative angles of the thigh and shank segments identified from ‘plug-in-gait bones’. Specifically, they are the three-dimensional rotation angles from the shank axes to the thigh axes.

The GRF magnitude and moment arm at instances of moment peaks were calculated for each plane of knee flexion and adduction to assess their relative contribution to the joint moments. In this process, GRF was projected on each plane determined from the knee joint axes. The moment arm was calculated from the orthogonal distance from the knee joint centre to the projected GRF.

Feature variables were the external first and second peak knee moments and the knee joint angles, moment arms, and GRF magnitude at instances of corresponding knee moment peaks; the external peak knee moments and knee angular impulses determined from the whole stance period; and spatiotemporal features such as step width, stride length, and walking speed. Feature variables were compared between the foot contact strategies through Wilcoxon signed rank test (because some of them failed the normality test) using SPSS version 24 for Windows (IBM Corp., Armonk, NY, USA).

## Data Availability

All data generated and/or analysed during this study are included in this published article.
